# Differences in Management and Outcomes in Atraumatic Splenic Rupture Compared to Traumatic Injury Following Blunt Abdominal Trauma

**DOI:** 10.3390/jcm13237379

**Published:** 2024-12-04

**Authors:** Katharina Rippel, Hannes Ruhnke, Betram Jehs, Mark Haerting, Josua A. Decker, Thomas J. Kroencke, Christian Scheurig-Muenkler

**Affiliations:** 1 Diagnostic and Interventional Radiology, University Hospital Augsburg, Faculty of Medicine, University of Augsburg, Stenglinstr. 2, 86156 Augsburg, Germany; katharina.rippel@uk-augburg.de (K.R.); bertram.jehs@uk-augsburg.de (B.J.); josua.decker@uk-augsburg.de (J.A.D.); christian.scheurig@uk-augsburg.de (C.S.-M.); 2 RIZ-Die Radiologen, Max-Josef-Metzger-Straße 3, 86157 Augsburg, Germany; hannes.ruhnke@gmx.de (H.R.); mark.haerting@t-online.de (M.H.); 3 Centre for Advanced Analytics and Predictive Sciences (CAAPS), University of Augsburg, Universitätsstr. 2, 86159 Augsburg, Germany

**Keywords:** splenic lacerations, AAST 2018, splenic artery embolization, atraumatic splenic laceration, traumatic splenic injury

## Abstract

**Background/Objectives**: To evaluate the differences in treatment and outcomes between traumatic and atraumatic splenic lacerations. **Methods**: This retrospective study included all patients with a diagnosis of splenic lacerations confirmed by computed tomography that presented from 01/2010 to 03/2023 at one tertiary hospital. The exclusion criteria included missing image data and death in the first 24 h due to extensive trauma. The etiology of the splenic laceration, demographic characteristics, and clinical parameters were recorded and evaluated as prognostic factors in therapy success and mortality. Subgroup analyses were undertaken according to the etiology of the splenic laceration and the primary treatment. The extent of splenic laceration was assessed by using the American Association for the Surgery of Trauma (AAST) score in its latest revision (2018). **Results**: Of all 291 enrolled patients (mean age 47 ± 21 years, 204 males), 50 presented with atraumatic splenic lacerations due to different underlying causes. The occurrence of moderate and high-grade laceration differed significantly between the atraumatic and traumatic study group (45/50 [90%] vs. 139/241 [58%], *p* < 0.001). Accordingly, the number of patients being treated conservatively differed greatly (20/50 [40%] vs. 164/241 [56%]), with a worse clinical success rate for atraumatic lacerations (75% vs. 94.5%). Atraumatic splenic injuries showed a higher conversion rate to surgery (2/20 [10%] vs. 2/164 [1%]). Despite the lower clinical success rate of splenic artery embolization (SAE) in atraumatic injuries (87% vs. 97%), the number of patients needing treatment for primary SAE in AAST 3 injuries was 14.1 in the traumatic population and only 4 in the atraumatic population. **Conclusions**: Atraumatic splenic injuries should not be treated as traumatic splenic injuries. An early upgrade to SAE or surgery should be considered for moderate splenic injuries, and they should be evaluated by an interdisciplinary team on a case-by-case basis. However, due to the underlying multimorbidity of patients with atraumatic splenic injuries, a higher mortality is to be expected.

## 1. Introduction

The spleen is the most commonly injured organ in the context of blunt abdominal trauma [1]. But, also, spontaneous splenic lacerations can occur in the context of an underlying disease, due to drugs, or without any discernible cause, although this occurs much less frequently [2,3,4,5]. Even cases where pregnancy caused atraumatic splenic ruptures have been described [6]. The loss of the spleen can subsequently increase the risk of infection and is associated with elevated mortality due to its importance to the human immune system and blood cell turnover [7]. There are clear guidelines in the management and treatment of traumatic splenic laceration in adults according to their severity [8,9]. The graduation of traumatic splenic lacerations is usually performed using the classification system of the American Association for the Surgery of Trauma (AAST) in its latest version from 2018 [10]. Non-operative management (NOM) in combination with a possible supplementary splenic artery embolization (SAE) has been established as an effective and safe organ-preserving strategy for traumatic lacerations [8,9,11,12]. Hemodynamically stable patients with an AAST score of 1 to 3 should be treated conservatively. Furthermore, especially in the treatment of children, a conservative treatment is often advisable [13,14]. In atraumatic cases, like a splenic rupture due to hemangioma, chronic myelomonocytic leukemia, or chronic pancreatitis, a partial or complete splenic embolization has also been described as a valid method of treatment [15,16,17]. Unlike in the treatment of traumatic splenic injuries, so far, there is no grading system specifically designed for atraumatic splenic lesions. Furthermore, there is little evidence regarding the safety and efficacy of NOM and SAE in cases of atraumatic splenic injury. Therefore, there are no standardized treatment paths for the treatment of atraumatic splenic injuries.

This monocentric retrospective study evaluated experiences in the management of patients presenting with splenic injury at a tertiary hospital over the last decade, with the aim of identifying the best treatment strategies for future cases, with a special focus on atraumatic splenic injuries.

## 2. Materials and Methods

### 2.1. Ethical Aspects

This study was conducted in accordance with the Declaration of Helsinki and approved by the Institutional Review Board of the Ludwig Maximilians University (protocol code 23-0528, 26 July 2023).

### 2.2. Patient Information

From 01/2010 to 03/2023, all patients that were diagnosed with splenic laceration were included in the study. The exclusion criteria included a diagnosis made solely by ultrasound, missing image data in the case of transfer from another hospital, and exitus letalis in the first 24 h due to extensive trauma to other organs. The etiology of the splenic laceration and epidemiological and clinical parameters of the patients were recorded and evaluated as possible prognostic factors in therapy success and mortality. Major complications after splenectomy and SAE according to the CIRSE Classification System were recorded [18]. Furthermore, subgroup analyses were undertaken according to the etiology of the splenic laceration and the primary treatment.

### 2.3. Etiology of Splenic Injury

Traumatic splenic injuries are by far the most common injuries. The rarer type of atraumatic splenic ruptures can be divided into those with idiopathic and pathological causes. In the case of an idiopathic splenic rupture, no underlying disease is detectable, there is no splenomegaly, and the histopathology of the spleen is physiological. Pathological splenic ruptures are based on neoplastic, infectious, inflammatory, non-infectious, drug- and treatment-related, or mechanical disorders. The main etiological groups, with common examples, are listed in Table 1.

Since appropriate classifications are only available for trauma-associated injuries, the extent of splenic laceration was assessed by using the widely utilized AAST (American Association for the Surgery of Trauma) score, in its latest revision from 2018, for all cases (Table 2) [10]. The evaluation was carried out by two radiologists experienced in the diagnosis and treatment of splenic lacerations in a consensus decision.

### 2.4. CT Protocols

In this retrospective study, all available CT scanners were used for imaging. The imaging protocols varied depending on the clinical presentation using different generations of CT scanners (Somatom Sensation 64, Somatom Definition Flash, Somatom Definition AS, Naeotom alpha, Siemens, Erlangen, Germany). In anticipation of an active bleed, patients underwent a multiphasic thoraco-abdominal or abdominal CT comprising a non-enhanced scan, an arterial and portal venous-phase scan, and an optional late-venous-phase scan. The decision was made by the attending radiologist at the time. If the CT was executed due to unclear abdominal pain, then a monophasic abdominal CT scan was performed in the portal venous phase. In the case of a polytraumatized patient, a native scan of the scull was acquired in addition to an arterial-phase scan of the neck and a portal venous-phase scan of the thorax and abdomen.

### 2.5. Treatment Options

Conservative treatment (NOM without SAE) was primarily based on monitoring the patient in the intensive care unit for usually 72 h with, if necessary, circulatory support, blood transfusion, and the correction of any coagulopathy. In higher-grade lacerations, SAE was performed as an adjunct to NOM, according to international guidelines. It was conducted at monoplane angiography units (Allura Integris until August 2017 and Allura Xper FD20 since September 2017, Philips, Amsterdam, The Netherlands) via unilateral transfemoral access. After selectively probing the celiac trunk, a digital subtraction angiography was performed to visualize the anatomy of the splenic artery, with a focus on early branching, elongation, the position of the dorsal and the great pancreatic artery, and any vascular pathologies or active hemorrhage within the spleen. Accordingly, the superselective probing of the splenic artery was performed using a diagnostic catheter or a coaxially advanced micro-catheter to reach the selected target point for embolization. Proximal SAE was defined as an embolization of the main stem of the splenic artery, distal to the dorsal pancreatic artery but proximal to the hilar branching. As a result of this approach, the arterial perfusion pressure onto the parenchyma was decisively reduced. Organ perfusion was usually maintained via collaterals, preventing infarction in the relevant organ and thus preserving the function of the spleen [20]. In selected cases with a distinct vascular pathology or a clear point of hemorrhage within the spleen, superselective distal embolization was performed. Embolization was performed by administering vascular coils, vascular plugs, or a combination of both until reaching complete stasis.

Technical success was defined as the successful placement of the embolization agents, while clinical success was defined as the clinical successful cessation of bleeding.

### 2.6. Study Objectives

The primary objective of this study was the comparison of treatment choices and effectiveness in traumatic and atraumatic splenic lacerations. This was achieved by calculating success rates and number-needed-to-treat values in both groups for different treatment options.

The secondary objective of this study was to share our experiences of treatment and outcomes in atraumatic splenic lacerations by publishing the largest current cohort.

### 2.7. Statistical Analysis

Statistical analysis was performed using Python (version 3.12.3). The normality of continuous variables was assessed using the Shapiro–Wilk test. Due to the non-parametric demographic and clinical data, as well as laboratory parameters, all data are presented as medians with corresponding ranges. Group comparisons were performed using the Chi-squared test for categorical variables and the Mann–Whitney U test for continuous variables. Statistically significant differences were assumed at *p*-values ≤ 0.05.

## 3. Results

In the study period, a total of 351 patients were diagnosed with splenic laceration. Three patients were excluded due to missing image data after transfer from another hospital. The diagnosis was made by ultrasound exclusively in 53 patients with only low-grade injuries and solely after blunt trauma. Four patients died within 24 h due to massive injury to the head or the thorax.

Of the remaining 291 patients, the vast majority of splenic lacerations diagnosed by CT were associated with blunt abdominal trauma (*n* = 241). The remaining 50 splenic lacerations were not caused by an external abdominal trauma. All 291 cases diagnosed by CT were therefore included in the present study, with special focus on the 50 atraumatic splenic lacerations.

A blunt splenic trauma is caused by a few typical mechanisms of injury. In contrast, there is greater heterogeneity in the mechanisms behind atraumatic splenic injury. Table 3 gives an overview of all aetiologies found in the atraumatic study population, including their respective frequencies.

Table 4 juxtaposes the grading of traumatic and atraumatic splenic injuries according to the AAST classification of 2018. The distribution of the severity of injury differed significantly among them (*p* < 0.001). Moderate-to-high-grade injuries (corresponding to AAST 3, 4, and 5) occurred in 90% of the atraumatic cases (45/50) compared to only 58% in traumatic injury (139/241). In particular, moderate injuries (AAST 3) were diagnosed more often (27/50 [54%] vs. 50/241 [21%]).

Table 5 shows detailed overall patient characteristics for traumatic and atraumatic splenic lacerations.

### 3.1. Traumatic Splenic Lacerations

**Figure 1:**
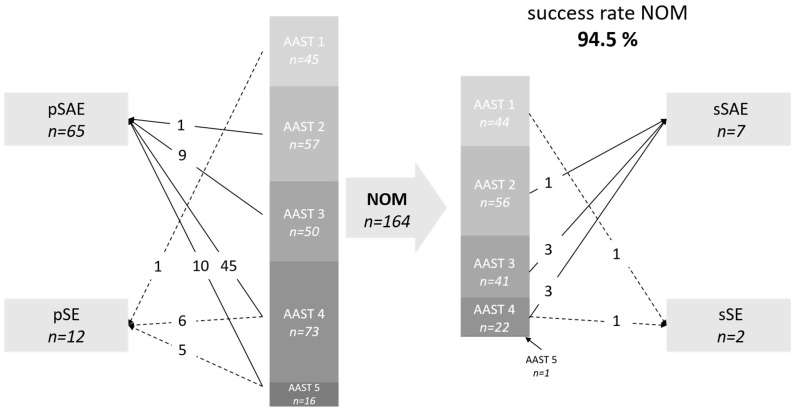
Primary and secondary lines of treatment for traumatic injuries. SAE, splenic artery embolization. SE, splenectomy; p, primary; s, secondary. AAST, American Association for the Surgery of Trauma. NOM, non-operative management.

Figure 1 shows the primary and secondary lines of treatment for traumatic injuries. In the traumatic population, a primary conservative treatment was conducted in 68% of cases (164/241), with a success rate of 94.5% (155/164). Only 2% (2/100) of the low-grade injuries (AAST 1 and 2) needed further treatment by either secondary SAE or surgery. With higher-grade injuries, the conversion rate rises significantly to 7.3% (3/41) for moderate injuries (AAST 3; *p* = 0.093) and 18.2% (4/22) for high-grade injuries (AAST 4; *p* = 0.009).

The number of primary splenic artery embolizations rose according to the severity of injuries. Overall, 65 patients were primarily treated, and 7 patients were secondarily treated, with SAE. The primary technical success rate was 100%, while the clinical success rate was 97% (70/72). Two patients showed further signs of bleeding and were then treated with a second SAE, with which the bleeding was successfully terminated. The reason for failure was an inadequate amount of embolic material in the first session of SAE and thus was avoidable.

When using the clinical success rate of 97%, the number needed to treat when comparing low-grade (AAST 1 and 2) and moderate (AAST 3) splenic injuries is 14.1, and it is 5.7 when comparing high-grade (AAST 4) and low-grade or moderate (AAST 1-3) splenic injuries.

One of the patients treated with SAE had to be converted to surgery (1/72; 1%). This was due to a splenic infarction with superinfection, which was not percutaneously drainable.

Major complications after SAE included persisting bleeding as mentioned above (*n* = 2), hematoma with superinfection (*n* = 3), necrosis of the pancreatic tail (*n* = 1), and extensive splenic infarctions (*n* = 6). This equals a major complication rate of 17% (12/72).

The overall mortality of 2.9% (7/241) was not associated with splenic injury but was mainly due to additional severe head trauma.

### 3.2. Atraumatic Splenic Lacerations

**Figure 2:**
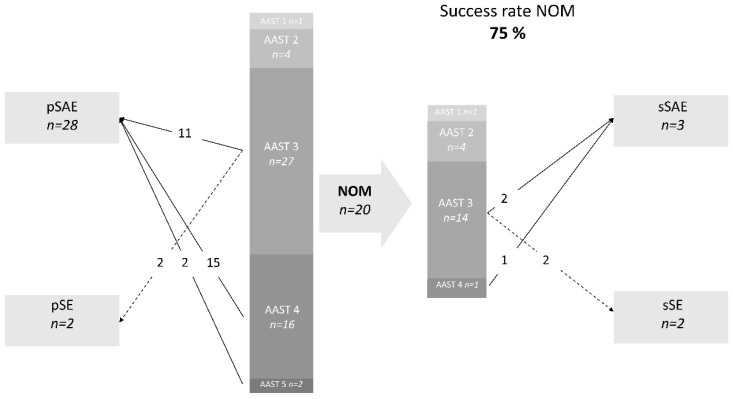
Primary and secondary lines of treatment for atraumatic injuries. SAE, splenic artery embolization. SE, splenectomy; p, primary; s, secondary. AAST, American Association for the Surgery of Trauma. NOM, non-operative management.

Figure 2 shows the primary and secondary lines of treatment for atraumatic injuries. Due to the larger number of moderate and high-grade injuries, less atraumatic injuries were treated conservatively (20/50), with a success rate of 75% (15/20).

A conversion to secondary SAE or surgery was observed for moderate atraumatic splenic lacerations (AAST 3) in 29% (4/14) of patients. Looking at the two largest subgroups of patients with atraumatic lacerations (injuries following medical procedures or those with an inflammatory cause), the picture is somewhat more differentiated. Three out of four patients with inflammatory etiology were deemed to be AAST grade 3 and had to be converted to secondary SAE or surgery after NOM because of clinical deterioration. In contrast, only one out of nine patients with injuries resulting from medical procedures and a moderate splenic laceration (AAST 3) needed a secondary conversion (11%). Actually, the outcome of patients with injuries resulting from medical procedures did not differ from those after trauma under otherwise-identical conditions (*p* = 0.671).

A large proportion of patients with atraumatic injuries was treated primarily by SAE (28/50; 56%), while another three patients were secondarily converted to SAE. Again, the technical success rate was 100%, while the clinical success rate was 87% (27/31). In four patients, there was a persistent bleeding; in two cases, this was due to the inadequate placement of embolic material, and in the other two cases, it was due to a newly established bleeding site in the inflammatory splenic lacerations. When calculating with a clinical success rate of 87%, the number needed to treat for moderate splenic injuries (AAST 3) was surprisingly low: only four patients.

The conversion to splenectomy was necessary in 5/31 (16%) cases after primary or secondary SAE. In one case, persistent postinterventional hemorrhage and progressive hemodynamic instability were responsible for the splenectomy. This was due to medical lysis applied because of a pulmonary embolism before SAE. In another case, splenectomy was performed in the setting of postinterventional laparotomy for persistent intraabdominal hemorrhage. Intraoperatively, however, this patient showed hemorrhage from the liver. Persistent active splenic bleeding was not detected. In three cases, primary treatment with SAE led to the cessation of the bleeding, but secondary perisplenic infection with insufficient drainage or the impossibility of draining the fluid retained percutaneously prompted a splenectomy. In one of these cases, the splenic injury was due to necrotizing pancreatitis; in another, it was due to the development of a pseudoaneurysm distal to the site of embolization in the context of a pronounced splenomegaly in acute myeloid leukemia. Furthermore, the third case was due to a splenic rupture in the context of Epstein–Barr virus infection.

Major complications included persisting bleeding as mentioned above (n = 4), hematoma with superinfection or infarction of the spleen (n = 4), and an ischemic ulceration of the stomach (n = 1). This equals an overall rate of major complications of 29% (9/31).

The mortality of the atraumatic study population was 18% (9/50), with two patients being treated conservatively (10%; 2/20) and seven patients being treated primarily by SAE (25%; 7/28). However, death was not directly associated with splenic rupture and hemorrhage in any of the cases. The patients died of their underlying diseases (non-Hodgkin lymphoma, acute myeloid leukemia, liver failure in liver cirrhosis, sepsis, postoperative course after mitral valve reconstruction, and one case with an unclear cause).

## 4. Discussion

Splenic laceration in the context of blunt abdominal trauma is a well-documented disease with clear diagnostic and therapeutic guidelines based on several studies with large patient populations [9,21,22,23,24]. Our study confirms the existing recommendations that low-grade and moderate traumatic splenic lacerations should primarily be treated conservatively by NOM. It shows a high rate of success with organ preservation (>90%). SAE should primarily be saved for high-grade splenic lacerations (AAST 4 and 5). However, diagnostic and therapeutic management and corresponding outcomes in atraumatic splenic injury have not been systematically addressed. This may be mainly due to the overall low incidence of the disease. So far, there are some smaller case series and even a review article, but most studies have focused on the etiology, and not the treatment, of atraumatic splenic ruptures [19,25,26].

The main findings of the present evaluation are as follows: (1) Patients with atraumatic splenic ruptures more often show higher-grade injury patterns at diagnosis. (2) Patients with atraumatic splenic rupture seem to have a significantly worse clinical outcome for the primary treatment strategy, which was chosen as an analogue to traumatic injuries (74% vs. 94.5%). (3) The subgroup of patients with moderate injuries (AAST 3), in terms of medical procedures, can be treated conservatively in comparison to trauma patients. Moderate splenic injuries with other atraumatic aetiologies show significantly more failure with conservative management. (4) Primary SAE is still a valid treatment option but proved to be less effective in atraumatic splenic rupture regardless of the etiology compared to traumatic patients (87% vs. 97%). (5) According to our experience, the higher overall mortality of patients with atraumatic injury seems not to have been directly associated with the splenic injury but may have been the consequence of their underlying disease, especially in the case of a malignant and inflammatory etiology. However, this needs to be confirmed with a larger cohort.

The rate of atraumatic causes among all splenic lacerations in the observation period was 14% (50/351), which is much higher than described by Liu et al. (3.2%) [27]. However, Liu et al. excluded iatrogenic causes of splenic lacerations from their study. In our study, this exclusion would also result in a considerably lower rate of 9% (29/330).

When comparing atraumatic and traumatic splenic lacerations, there is a clear difference in the distribution of severeness. Overall, 90% of the patients in the atraumatic group were diagnosed with a moderate or high-grade splenic laceration (AAST 3, 4 and 5; 45/50), while only 58% (139/241) of patients presenting with a traumatic splenic laceration were deemed as having moderate or high-grade lacerations. Similar distributions of traumatic and atraumatic splenic lacerations were shown in other studies [24,28]. The lower prevalence of severe injuries for traumatic lacerations is probably adequately explained by the systematic diagnostic workup of trauma patients, whereby many clinically silent lacerations are discovered and documented. In contrast, atraumatic patients are mainly diagnosed in the symptomatic stage, when the cause of a respective upper abdominal complaint is specifically investigated. The comparatively long time until diagnosis in atraumatic splenic lacerations as shown in other studies supports this thesis [19]. While traumatic splenic lacerations mostly affect people with a low pre-existing morbidity, e.g., in traffic accidents, patients with atraumatic splenic lacerations are often older and present with relevant comorbidities [19,24]. This is also shown by the most frequent causes in the present study: medical procedures or intraoperative injuries, inflammatory processes such as pancreatitis, or treatment with anticoagulants. The high number of infectious causes for atraumatic splenic ruptures described in one review by Aubrey-Bassler et al. was caused by malaria, which is very uncommon in this part of the world [19].

The number of patients primarily being treated conservatively in the atraumatic patient group (20/50; 40%) was slightly lower than in the traumatic study group (164/241; 56%). Accordingly, the number with primary SAE was considerably higher in the atraumatic study group (28/50; 56% vs. 65/241; 27%), which is very likely due to the higher grading. The decision toward primary SAE was made according to the generally approved consensus for the treatment of traumatic splenic lacerations [11]. According to recommendations for the treatment of traumatic splenic lacerations, SAE is preferred in the presence of higher-degree splenic injuries (AAST 4 and 5), whereas low-grade and moderate injuries (AAST 1-3) can be satisfactorily treated conservatively to a large extent [29]. However, considering the poor clinical success rate of NOM (75%) in atraumatic cases of moderate atraumatic injuries and the surprisingly low number needed to treat of only four patients in this study, there seems to be a necessity to rethink the current standard of practice. The results also showed a more generous indication for endovascular or surgical therapy in patients with atraumatic splenic laceration in the AAST 3 subgroup, in contrast to the recommendation in traumatic splenic lacerations. This takes into account the lower clinical success rate of SAE in these cases.

However, this is only part of the problem in terms of adequately treating these patients according to their stage. The clinical outcome after SAE in higher-grade injuries (AAST 4 and 5) in atraumatic patients was worse than in trauma patients. This was not related to splenic rupture. Among the five patients requiring secondary surgery, one showed persistent bleeding with a progressive hemodynamic instability due to applied medical lysis before the SAE because of pulmonary embolism, and three showed secondary abscesses during the postinterventional infarction of the spleen. The latter may be explained due to the limited vascular collaterals after inflammatory processes in the pancreas and a consecutively higher risk of infarction and superinfection after the proximal occlusion of the splenic artery.

The secondary splenectomy rate of 15% (7/48) due to superinfection or continuous bleeding in the atraumatic group seems rather high compared to the 1% (3/279) of the traumatic group. Low conversion rates of 3–18% have been described in other studies on traumatic splenic embolizations [12,22,30]. Again, this is probably attributable to the severeness of splenic lacerations at the time of diagnosis, the higher risk of superinfection, and the underlying diseases.

No clear statement can be made in relation to the present study about the course of higher-degree atraumatic splenic injuries under conservative therapy, because only one patient with a fourth-degree splenic laceration due to a postoperative abscess was treated in a primarily conservative way, and persistent bleeding led to secondary conversion to SAE. However, in the light of the results of this study, it is highly unlikely that better results would be observed with conservative treatment in high-grade injuries. In particular, in the presence of anticoagulation, which may not be sufficiently antagonized with the corresponding indication, or in the presence of local inflammation, a protective SAE seems to be reasonable even in the absence of active bleeding and in cases of minor splenic injury in individual cases. In these situation, close consultation between interventionalists, surgeons, and emergency physicians is required to assess the individual risk profile of the patient.

Finally, the management of atraumatic splenic injuries has a treatment-independent worse outcome compared to traumatic splenic injuries. The indication for endovascular or surgical therapy should be made more generously in the context of the individual etiology, especially in the case of inflammatory, and thus potentially progressive, intra-abdominal processes. In many cases, however, the patient’s underlying disease remains the limiting factor in terms of outcomes. SAE can also be considered an effective therapy in the treatment of atraumatic splenic laceration, even though the risk of postinterventional infarction with possible secondary infection seems to be more important than in traumatic organ injuries.

The limitations of this study are mainly linked to its retrospective character. Obviously, no form of randomization was possible. Due to the long period of patient inclusion (13 years), the treatment of choice changed over time. Furthermore, the numbers in both groups varied greatly, which is due to the rarity of atraumatic splenic injuries.

## 5. Conclusions

Atraumatic splenic injuries should not be treated the same as traumatic splenic injuries. An early upgrade to SAE or surgery should be considered for moderate atraumatic splenic injuries and evaluated by an interdisciplinary team on a case-by-case basis. However, due to the underlying multimorbidity of the patients with atraumatic splenic injuries, a higher mortality is to be expected.

## Figures and Tables

**Table 1:** Common causes of atraumatic splenic ruptures [19].

Major Etiological Groups	Common Examples
Haematologic disorders	Malignant hematologic disorders Non-malignant hematologic disorders
Neoplastic disorders	Primary neoplastic disorders Secondary metastatic
Infectious disorders	Viral Bacterial Protozoal Fungal
Inflammatory, non-infectious disorders	Local inflammatory disorders Amyloidotic disorders Vascular disorders
Genetic disorders	Hematological disorders Storage diseases
Autoimmune disorders	Rheumatoid arthritis Systemic lupus erythematosus
Drug-related cause	Anticoagulation Thrombolytic therapy Granulocyte colony-stimulating factor Hemodialysis
Medical procedures	Endoscopy Surgery ESWL (extracorporeal shock wave lithotripsy)
Mechanical disorders	Pregnancy-related cause Congestive splenomegaly

**Table 2:** Assessment of the extent of splenic injury according to AAST (American Association for the Surgery of Trauma) classification (2018) [10].

Grade	AAST 2018 CT Findings	
	Hematoma/Vascular Injury	Laceration
1	Subcapsular < 10% of surface area	Parenchymal laceration < 1 cm deep or Capsular tear
2	Subcapsular 10–50% of surface area or Intraparenchymal hematoma < 5 cm	Parenchymal laceration 1–3 cm deep
3	Subcapsular > 50% of surface area Or ruptured subcapsular or parenchymal hematoma ≥5 cm	Parenchymal laceration >3 cm deep
4	Splenic vascular injury * or active bleeding ** within splenic capsule	Segmental or hilar vessel involvement with major devascularization (>25%) of spleen
5	Splenic vascular injury * with active bleeding ** beyond the spleen	Shattered spleen

* Vascular injury is defined as a pseudoaneurysm or arteriovenous fistula and appears as a focal collection of vascular contrast that decreases in attenuation with delayed imaging. ** Active bleeding from a vascular injury presents as a vascular contrast, focal or diffuse, that increases in size or attenuation in the delayed phase. Advance one grade for multiple injuries up to Grade 3.

**Table 3:** Etiology of atraumatic splenic ruptures.

Atraumatic	Cause of Injury	*N*
Medical procedures	Intraoperative injury Colonoscopy Reanimation Thoracic drainage Gastroscopy Transgastric drainage Splenic punction	8 5 2 2 2 1 1
Inflammatory disorders	Acute pancreatitis Local peritonitis Septic embolism	6 1 1
Drug-related causes	Anticoagulation	5
Hematolgical disorders	Myelodysplatic syndrome Myelofibrosis Acute myelogenous leukemia	1 1 1
Mechanical disorders	Portal hypertension Splenic vein thrombosis	1 1
Infectious disorders	Infectious mononucleosis	2
Neoplastic disorders	Malignant infiltration	1
Unknown		8

**Table 4:** Grading of atraumatic and traumatic splenic laceration according to the AAST grading (2018) [10].

	Atraumatic	Traumatic
AAST 1	1 (2%)	45 (19%)
AAST 2	4 (8%)	57 (24%)
AAST 3	27 (54%)	50 (21%)
AAST 4	16 (32%)	73 (30%)
AAST 5	2 (4%)	16 (7%)
Σ	50 (100%)	241 (100%)

**Table 5:** Patient characteristics for atraumatic splenic lacerations and subgroups due to etiology.

	Overall—Traumatic	Overall—Atraumatic	*p*-Values
Number of patients	241	50	
Age in years *	41 (7–97)	65 (22–88)	<0.001
Gender m/f	171/70	33/17	0.598
Hospital stay in days *	14 (1–62)	21 (1–100)	0.216
ICU stay in days *	5 (0–54)	5 (0–23)	0.414
Hemodynamic instability at time of diagnosis (%)	38 (16)	21 (42)	<0.001
Conservative management (%); successful (%)	164 (68); 155 (95)	20 (40); 15 (75)	<0.001 0.0078
Primary SAE (%); successful (%)	65 (27); 63 (97)	28 (56); 24 (86)	<0.001 0.119
Primary surgery (%)	12 (5)	2 (4)	0.765
Overall mortality (%)	7 (3)	9 (18)	<0.001

* Median (range or percentage); ICU—intensive care unit; SAE—splenic artery embolization.

## Data Availability

The original contributions presented in this study are included in the article. Further inquiries can be directed to the corresponding author.
